# Role of MicroRNAs in the Regulation of Subcutaneous White Adipose Tissue in Individuals With Obesity and Without Type 2 Diabetes

**DOI:** 10.3389/fendo.2019.00840

**Published:** 2019-12-05

**Authors:** O. Brovkina, A. Nikitin, D. Khodyrev, E. Shestakova, I. Sklyanik, A. Panevina, Iurii Stafeev, M. Menshikov, A. Kobelyatskaya, A. Yurasov, V. Fedenko, Yu Yashkov, M. Shestakova

**Affiliations:** ^1^Endocrinology Research Centre, Moscow, Russia; ^2^Federal Research and Clinical Center, Federal Medical-Biological Agency of Russia, Moscow, Russia; ^3^Pulmonology Research Institute, Federal Medical-Biological Agency of Russia, Moscow, Russia; ^4^National Medical Research Centre for Cardiology, Moscow, Russia; ^5^Engelhardt Institute of Molecular Biology, Russian Academy of Sciences, Moscow, Russia; ^6^Central Clinical Hospital and Polyclinic, Moscow, Russia; ^7^Institute of Plastic Surgery and Cosmetology, Moscow, Russia; ^8^Center of Endosurgery and Lithotripsy, Moscow, Russia

**Keywords:** metabolically healthy obesity, microRNA, RNAseq, metalloproteinases, SMAD4, RUNX2, type 2 diabetes

## Abstract

Obesity is a high-risk factor for such comorbidities as cardiovascular disease, several types of cancer, and type 2 diabetes; however not all individuals with obesity have such complications. Approximately 20% of individuals with obesity are metabolically healthy. This study focused on differences between obese individuals with and without type 2 diabetes (T2D+ and T2D–, respectively) on the transcriptome level. Subjects included were 35 T2D– patients with obesity and 35 T2D+ patients with obesity with the same body mass index (BMI). The study was based on the transcription analysis of mRNA and microRNAs (miRs) by RNAseq. In the first step, we performed RNAseq of miRs, in the second step, we analyzed only those mRNA, which appeared targets for significant miRs from the first step. All RNAseq results were validated by qPCR. There were seven miRs differently expressed with adjusted *p*-value <0.1, which were confirmed by qPCR. Five among them: miR-204-5p, miR125b-5p, miR-125a-5p, miR320a, miR-99b—were upregulated in T2D+ patients with obesity, while only two miRs, miR-23b-3p, and miR197-3p, were increased in T2D– patients with obesity. These seven miRs target two groups of genes: matrix metalloproteinases and TGFβ signal pathway genes. According to the results of transcriptome analysis, the main difference between T2D+ and T2D– patients with obesity was in adipogenesis and fibrosis regulation by matrix metalloproteinases and SMAD4-RUNX2 signal cascade. Based on the data about transcription profiles of both groups, we suggested that the process of fibrosis in T2D+ patients with obesity is more pronounced than in T2D– patients with obesity.

## Introduction

The prevalence of obesity has increased worldwide over the past few decades (1). Obesity is associated with type 2 diabetes (T2D), cardiovascular diseases (hypertension, dyslipidemia, coronary heart disease, heart failure, stroke), neurological disease, cancer, and influence on the immune system and fertility (2, 3). One of the frequently used methods to define obesity is body mass index (BMI). By this classification, individuals with obesity have BMI values of more than 30 kg/m^2^. Other important characteristics of obesity are the percentage and content of adipose tissue. The adipose tissue is composed of white adipose tissue (WAT) and the brown adipose tissue (BAT), which have different morphology, distribution, gene expression, and function (4). WAT is composed of adipocytes surrounded by the stromal vascular fraction. WAT depots can be located in the abdominal cavity; in this case, it will be visceral fat, which was considered to be more metabolically active. Subcutaneous WAT depots are located underneath the skin. It serves as thermal isolation and perform mechanical and structural functions. In cases of obesity, the WAT amount may reach up to 50% of body weight. Recent studies show that WAT plays an important endocrine and immune role and affects the functionality of cells and tissues all over the body (5). Many individuals with obesity have T2D due to the presence of severe insulin resistance. However, there is a group of patients with long-standing obesity who do not develop T2D. To date, there are no clear mechanistic understandings of development insulin-sensitive obesity (6). One of the assumed reasons is a genetic ground (7). Therefore, the studies which include gene expression analysis in T2D+ and T2D– patients with obesity provide valuable information for the prevention of cardiovascular and metabolic complications in individuals with obesity. Considering that WAT plays a role both in lipid and glucose regulation, Das et al. identified the difference in RNA transcripts of T2D+ and T2D– patients with obesity (8). In that study, the most difference in gene expression was observed for metallopeptidase 9 and osteopontin (MMP9 and SPP1) as upregulated genes and for cytoplasmic protein NDRG4 and subunit 3 in DNA replication complex GINS (GINS3) as downregulated genes.

Up to 60% of gene expression can be regulated by microRNAs (miRs) (9). MiRs are endogenous, short, non-coding molecules with a length of 20–25 nucleotides, which can bind the complementary sequence of mRNA and inhibit the translation. The growing number of publications suggests that miRs play a pivotal role in adipose tissue by targeting adipocytes metabolism and insulin signaling (9). Jordan et al. showed that miR-143 had been a positive regulator of human adipocyte differentiation, acting via ERK5 signaling (10). Other studies revealed that miR-27a and miR-130a inhibited adipocyte differentiation through PPARγ downregulation (11, 12). Thomou et al. have recently described adipose tissue as a primary source of circulating exosomal miRs (13). Circulating exosomal miRs derived from fat may act as regulators of whole-body metabolism and mRNA translation in other tissues (14–16). The definition of the miR profile of adipose tissue in patients will help not only in diagnostics purposes but also in therapeutic strategy. This strategy may include inhibition of certain miRs, as well as increasing miRs level with miR mimics (9, 17).

The aim of the present study was to identify differences in miR and mRNA expression between T2D+ and T2D– patients with obesity. We started from RNAseq analysis of miRs; next, we analyzed only those mRNA, which appeared targets for significant miRs from the first step.

## Subjects and Methods

### Patients

The study was approved by the Endocrinology Research Center ethics committee (protocol No. 9, 10.09.2017). Written informed consent was obtained from all the volunteers.

Seventy patients (35 in T2D– group and 35 in T2D+ group) participated. All patients had a long duration (>15 years) of morbid obesity (BMI > 35 kg/m^2^). Subjects <18 years old and with any other type of diabetes, pregnancy, or cancer were excluded from the study. T2D+ patients were diagnosed before including in the project on the basis of the Endocrinology Research Center. Obese T2D+ individuals were prescribed diabetes medications: 22.8% of patients received monotherapy, 42.9%- two-component therapy, 34.3%- three-component (Table S1).

### Clinical Characteristics

Fasting glucose was measured with Architect c4000 clinical chemistry analyzer (Abbott Diagnostics, Abbotpark, IL, USA) using standard kits offered by the manufacturer. Glycated hemoglobin (HbA1c) was assessed with high-performance liquid chromatography (D-10 Hemoglobin Testing System, BioRad, France). The lipid profile was measured by Architect c4000 analyzer (Abbott Diagnostics, Abbotpark, IL, USA).

### Insulin Resistance

Insulin resistance was measured by two methods: the HOMA-IR calculator and the hyperinsulinemic-euglycemic clamp test.

The HOMA-IR was calculated by the formulae:

$$\begin{array}{l} \text{Insulin resistance}=\text{FI}\times \text{G}/22.5, \end{array}$$

where FI = fasting insulin μIU /ml. And G = fasting glucose (mmol/l).

The classic DeFronzo hyperinsulinemic-euglycemic clamp test was used to assess insulin resistance (IR) (18). Insulin was continuously infused at 100 μU/mL to inhibit systemic insulin secretion by the pancreas and liver. Simultaneously 20% glucose was intravenously infused to reach normal blood level that was subsequently maintained by a controlled infusion rate using Infusomat FMS volumetric infusion pump (B. Braun, Germany). The insulin infusion rate was set to 1 mU/kg/min by Perfusor compact syringe pump (B. Brown, Germany). Blood glucose was measured every 5–10 min with One Touch Verio Pro+ glucometer (Life Scan, Switzerland). The euglycemic option was chosen in order to mitigate the effects of hyperglycemia on glucose uptake. The target blood glucose levels were 5.1–5.6 mmol/L. If blood glucose has decreased glucose infusion was accelerated, and vice versa. In about 120–180 min, a dynamic equilibrium was achieved, meaning that the glucose infusion rate was equal to glucose uptake by the tissues. The glucose infusion rate at a dynamic balance over 30–40 min was considered to be the glucose uptake by tissues. It used to calculate glucose consumption (M value) as a mean of 6–8 glucose infusion rate readings per kg of body mass per minute. The results were expressed as M values (mg/kg/min) and classified as follows: M = 0–2 severe IR; M = 2–4 moderate IR; M = 4–6 mild IR; M > 6 no IR. Subcutaneous WAT biopsies were obtained from patients during bariatric surgery after an overnight fast.

### RNAseq Analysis

The tissue samples from patients were immediately placed in lysis buffer (50–80 mg in 1 ml QIAzol Lysis Reagent (Qiagen, Germany) and homogenized by a TissueLyser LT (Qiagen, Germany). Total RNA isolation from adipose tissue was carried out by an Rneasy Lipid Tissue Mini Kit (Qiagen, Germany) on the automatic QIAcube station according to the manufacturer protocol. To prevent degradation, we added 1 unit of RiboLock Rnase Inhibitor (Thermo Fisher Scientific, USA) per 1 μL of RNA solution. The concentration of total RNA in the aqueous solution was evaluated on a NanoVue Plus spectrophotometer (GE Healthcare, USA).

MiRs expression was then analyzed by sequencing on Illumina NextSeq 500 (Illumina, USA). The libraries were prepared by the Illumina TruSeq Small RNA Library Prep Kit following the manufacturer standard protocols. Strand-specific sequencing was performed for a total of 72 samples (31 samples from T2D– patients with obesity and 31 samples from T2D+ patients with obesity). The bioinformatics processing was as follows: adaptors were trimmed with Cutadapt; the resulting FASTQ files were then mapped onto the human genome (GRCh37 assembly) with bowtie2. FastQC was used as a tool to visualize different quality control measurements.

For each sample, sequences were annotated using human pre-miRNA and mature miRNA databases provided by the miRBase (http://microrna.sanger.ac.uk/sequences/) in SeqBuster. The resulting count data were analyzed in DESeq2 to obtain differentially expressed miRNAs. Only log_2_-fold changes with an adjusted *p*-value of 0.10 were considered significant. We used TargetScan, Diana-TarBase v8, and mirPath v.3 for sequence-based target prediction.

Strand-specific mRNAs sequencing was performed by Illumina NextSeq 500. The libraries were prepared by the Illumina TruSeq RNA Library Prep Kit following the manufacturer standard protocols. Sequencing was done for a total of 48 samples (24 samples from T2D– patients with obesity and 24 samples from T2D+ patients with obesity). The bioinformatics processing was as follows: adaptors were trimmed with Cutadapt; the resulting FASTQ files were then mapped onto the human genome (GRCh37 assembly) using STAR. HTSeq was used to count the number of reads, and resulting count data were analyzed in edgeR to obtain differentially expressed mRNAs.

The miRNet platform was used to integrate miRs and their target genes.

Pheatmap (R package) was used for the heatmap diagram; only log_2_-fold changes with an adjusted *p*-value of 0.10 were considered significant.

### RT-PCR Data Analysis

Reverse transcription-quantitative PCR (RT-qPCR) of miRs in 70 samples was carried out by a TaqMan Advanced miRNA cDNA Synthesis Kit (Thermo Fisher Scientific, USA) and TaqMan Advanced miRNA Assays (Thermo Fisher Scientific, USA), in 96-well plates on the StepOnePlus PCR System (Applied Biosystems, USA), according to the manufacturer protocols. All samples were normalized to hsa-miR-191 levels and spike-in control cel-miR-39-3p. RT-qPCR assays were performed to validate the DESeq2 differential expression results for miRNAs with mean of normalized counts > 100.

A two-step qRT-PCR of mRNA in 70 samples was carried out using a High-Capacity RNA-to-cDNA Kit (Thermo Fisher Scientific, USA) and Custom TaqMan Array 48 Plus plates (Thermo Fisher Scientific, USA), in 96-well plates on the StepOnePlus PCR System (Applied Biosystems, USA), according to the manufacturer protocol. All samples were normalized to GUSB and levels of GAPDH, HPRT served as secondary internal controls.

Data analysis was performed by SDS software (version 2.3. Applied Biosystems). A *P* value < 0.05 was considered statistically significant. In order to account for multiple comparisons, a correction for the false discovery rate (Q values <0.10) was calculated using the Benjamini-Hochberg adjustment.

## Results

According to the design of the project, subjects had no significant difference in BMI, but a comparison of HOMA-IR and M-index revealed substantial differences between the two groups of patients. Patients characteristics are shown in Table 1. Obese T2D+ group had severe IR comparing to obese T2D– group according to both clamp test and HOMA-IR (Table 1). There was also a significant difference in triglycerides level, where T2D+ patients with obesity had higher amounts.

**Table 1:** Patient characteristics (Me [Q25. Q75]).

	**T2D– patients with obesity *n* = 35**	**T2D+ patients with obesity *n* = 35**	***p***
Sex (m:f)	10:25	11:24	
Age, years	44.5 [38.5; 49.0]	48.5 [43.0; 54.0]	0.61
BMI, kg/m^2^	43.02 [40.83; 46.67]	41.80 [37.78; 44.75]	0.109
M-index, mg/kg/min	4.13 [3.17; 5.08]	1.52 [0.98; 2.23]	**<0.001**
HOMA-IR	4.78 [2.26; 5.80]	10.25 [6.01; 18.81]	**0.026**
Fasting blood glucose, mmol/l	5.16 [4.87; 5.78]	8.89 [7.27; 12.52]	**<0.001**
HbA1c, %	5.40 [5.30; 5.78]	8.00 [7.20; 8.96]	**<0.001**
Total cholesterol, mmol/l	5.43 [4.77; 6.15]	5.27 [4.60; 6.23]	0.919
Cholesterol (HDL), mmol/l	1.12 [1.00; 1.49]	1.14 [0.99; 1.32]	0.853
Cholesterol (LDL), mmol/l	3.28 [2.92; 3.74]	3.45 [2.91; 4.35]	0.217
Fasting triglycerides, mmol/l	1.55 [1.02; 2.13]	2.76 [1.96; 3.46]	**<0.01**

*The bold values are values which had statistically significant difference*.

The miRNA-seq generated an average of 9.2 million single-end reads per sample, a total of 207 mRNA genes appeared to be DE with a significance threshold of adjusted *p*-value ≤ 0.05 and fold-change ≥ 2.0 After the removal of low-quality reads and adaptor sequences, the mean yield of clean reads was 8.73 million (range: 6.13–15.79 million). Length distribution analysis of all samples revealed an expected peak at 20–24 nts, consistent with the size of miRs, while there was another one peak at 32–33 nts. There were 345–679 known miRs annotated in samples, most of the miRs were detected in all samples. All sample-specific miRs were lowly expressed (count <10), suggesting that these are of limited biological meaning.

We analyzed the differential expression of miRs by comparing the miR in both groups. There was seven miRs differently expressed with adjusted *p*-value < 0.1, which were confirmed by qPCR (Table 2, Table S2). Five among them: miR-204-5p, miR125b-5p, miR-125a-5p, miR320a, miR-99b—were downregulated in T2D– patients with obesity. MiR-23b-3p and miR197-3p were hyper expressed in T2D+ patients with obesity.

**Table 2:** Differential expression of significant validated miRNAs (comparison T2D– vs. T2D+ groups, adjusted *p*-value <0.05 and mean of normalized counts > 10).

**NGS**					**qPCR**			**Predicted targets**
**MicroRNA (miR) symbol**	**Mean of normalized counts (rounded)**	**log_**2**_-FC**	***p*-value**	**Adjusted *p*-value**	**log_**2**_-FC (95% confidence interval)**	***p*-value**	**Adjusted *p*-value**	
hsa-miR-23b-3p	429	1.31	1.24E-05	0.00016305	2.75 (2.01–3.56)	<0.001	0.012	TUSC7 RUNX2 TGIF1 PTEN VHL MET NOTCH2 TMEM64 TNFAIP8
hsa-miR-99b-5p	10,114	−1.01	0.0002011	0.0013042	0.42 (0.29–0.64)	<0.001	0.01	NOX4 IGF1R RAVER2 MTOR ARID3A
hsa-miR-125a-5p	27,497	−1.36	3.41E-08	1.33E-06	0.51 (0.38–0.75)	<0.001	0.001	MMP11 SMAD4 TNFRSF10B VEGFA IFNG E2F3 MAPK14
hsa-miR-125b-5p	4,621	−1.02	0.00021621	0.0013922	0.46 (0.31–0.65)	<0.001	0.01	MAPK14 MMP2 MMP13 ERBB3 ERBB2 BMF MMP26 MAP3K11 IGF2 BMPR1B SMAD4
hsa-miR-197-3p	7,053	1.01	9.13E-06	0.00012774	1.47 (0.92–1.98)	<0.001	0.02	BMF FOXJ2 MAPK1 FBXL13
hsa-miR-204-5p	1,107	−1.67	1.93E-05	0.00021278	0.18 (0.08–0.28)	<0.001	<0.001	IL11 ANKRD13A IGFBP2 HMX1 BDNF RUNX2 VIM CREB5 SOX4 CDX2 ALPL TGFBR1 SOST FOXC1 SMAD4 SMAD6 MMP9
hsa-miR-320a	4,127	−1.15	1.95E-05	0.00021278	0.50 (0.34–0.77)	<0.001	0.015	FOXM1 MMP9 RAB14 MAPK1 PTEN RUNX2 NOD2 FBXL13

*FC (fold-change)—the log-ratio of a gene expression values in two different conditions*.

The results of RNAseq data allowed us to divide up- and downregulated genes in obese T2D+ and T2D– patients. But no specific patterns were found in these groups: both groups of patients had upregulated genes linked to inflammation processes and cell proliferation (Figure S1).

The RNA-seq generated an average of 47 million single-end reads per sample, a total of 552 mRNA genes appeared to be DE with a significance threshold of adjusted *p*-value ≤ 0.05 and fold-change ≥ 2.0. For subsequent RNAseq analysis, we selected genes, which had intersections with previously revealed significant miRs. Validation by qPCR analysis confirmed only six genes whose expression showed a significant difference: MMP9, MMP2, MMP26, MMP11, SMAD4, RUNX2 (Table 3, Table S3). All these genes were upregulated in T2D– patients with obesity. The most genes appeared to be members of the zinc-metalloproteinases family involved in the degradation of the extracellular matrix: MMP9, MMP2, MMP26, MMP11. The remaining part of the genes belonged to the TGFβ/BMP signaling pathway: SMAD4 and RUNX2.

**Table 3:** Differential expression of significant validated mRNAs (comparison obese T2D– vs. obese T2D+ groups, adjusted *p*-value <0.05) filtered by differently expressed miRNA from Table 2.

**NGS**					**qPCR**			**Known miRNAs**
**Gene symbol**	**log_**2**_-FC**	**log_**2**_-CPM**	***p*-value**	**Adjusted *p*-value**	**log_**2**_-FC (95% confidence interval)**	***p*-value**	**Adjusted *p*-value**	
MMP9	1.9675	5.0787	1.0279e-30	4.6098e-28	1.92 (1.51–2.31)	<0.001	0.004	hsa-mir-211-5p hsa-mir-320a hsa-mir-204-5p hsa-mir-491-5p hsa-mir-132-3p
MMP2	2.0971	6.9353	1.6109e-49	1.8509e-46	1.29 (1.11–1.46)	0.002	0.042	hsa-mir-29a-3p hsa-mir-29b-3p hsa-mir-29c-3p hsa-mir-491-5p hsa-mir-130b-3p hsa-mir-125b-5p
MMP26	2.0712	6.8292	8.4676e-45	7.5949e-42	1.29 (1.11–1.46)	0.002	0.042	hsa-mir-125b-5p hsa-mir-124-3p
MMP11	2.85	5.4909	2.7054e-75	7.2798e-72	1.44 (1.22–1.67)	<0.001	0.007	hsa-mir-135a-5p hsa-mir-125a-5p hsa-mir-139-5p
SMAD4	3.0695	8.8106	6.8842e-90	2.3155e-86	3.24 (1.98–4.50)	0.002	0.040	hsa-mir-125a-5p hsa-mir-125b-5p hsa-mir-125b-5p hsa-mir-199a-5p hsa-mir-26a-5p
RUNX2	1.297	4.6497	2.011e-17	2.7329e-15	1.5 (1.29–1.70)	<0.001	0.001	hsa-mir-204-5p hsa-mir-205-5p hsa-mir-23b-3p hsa-mir-320a hsa-mir-335-5p

*FC (fold-change)—the log-ratio of a gene expression values in two different conditions*.

Significant miRs, which we found in the first step of this study, are connected predominantly with processes of gene expression and translation (Table S4). That is expected and obvious, but the connection with cellular responses to stress and activation of matrix metalloproteinases is interesting and can underlie the differences between obese T2D+ and T2D– patients.

**Figure 1:**
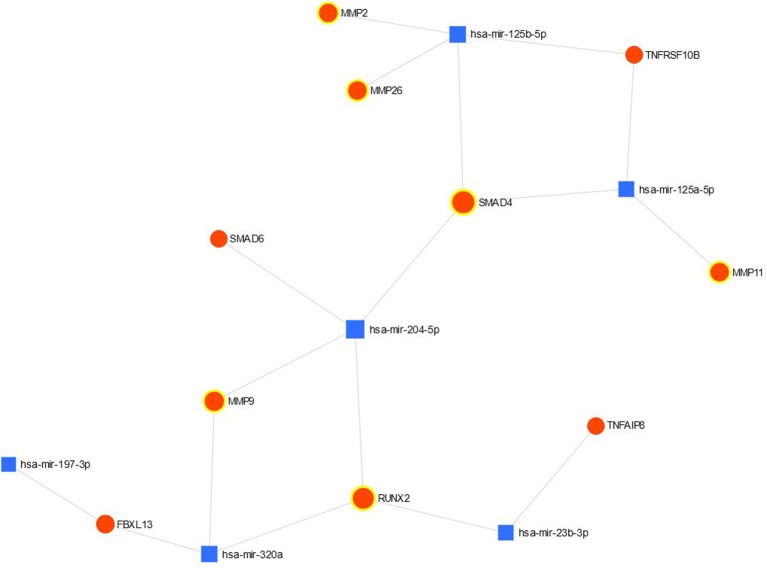
The network view of miRNA-gene interaction validated up- and down-regulated genes are highlighted.

The connection between validated significant miRs and their targets is shown in Figure 1. MiR- 99b-5p is absent on the figure since this miR had no validated targets with the significant differences in the expression. Most of the targets belong to miR-204-5p and miR125b-5p.

## Discussion

The underlying hypothesis of this study was that miRs can play a crucial role in developing glucose metabolism disturbance in patients with similar obesity history and comparable BMI.

In our study, expression of seven miRs was significantly different between obese T2D+ and T2D– patients. One of these miRs, miR-99b-5p, was overexpressed in T2D+ patients with obesity, but we did not observe any significant changes in the expression of target genes for this miR. MiR-99b-5p is a member of miR-125a-let-7e cluster, and according to recent studies, plays a role in the differentiation, migration, and proliferation of cancer cells (19). Wang et al. showed an association of miR-99b with tumor pathogenesis (20). Down-regulation of miR-99a/b was observed in human cancers, suggesting that miR-99a/b may function as tumor suppressors. This effect authors explained by such the fact that IGF-1R (insulin-like growth factor 1 receptor) is a direct co-target of miR-99b-5p. For our study, this link is relevant since the IGF-1R is dependent on BMI and plays a role in the regulation of β-cell mass, insulin secretion, and the regulation of insulin sensitivity. Decreased IGF-1R expression level contributes to insulin sensitivity. We suggest, that in T2D+ patients with obesity, overexpressed miR-99b-5p may present a negative feedback mechanism to balance hormone disorder (21).

MiR-197-3p and miR-23b-3p were downregulated in T2D+ patients with obesity; thus we could expect increased expression of their targets, TNFAIP6, RUNX2, and FBXL13, in these patients. But in fact, we did not observe any difference in the expression of target genes between the patients. Moreover, on the contrary, RUNX2 had lower expression in T2D+ patients with obesity compared with T2D– patients with obesity. Recent studies show that miR-23b is a very potent post-transcriptional regulator of growth and differentiation during cell cycle progression (22). Decreased level of this miR in T2D+ patients with obesity may indicate dysregulation of normal tissue homeostasis.

Four miRs, miR-204-5p, miR-125b-5p, miR-125a-5p, miR-320a, were overexpressed in T2D+ patients with obesity. These miRs are associated with matrix metalloproteinases (MMP-2, -9, -11, -26) and TGFβ/BMP signaling components (SMAD4, RUNX2). As expected, target genes had decreased levels of expression.

Matrix metalloproteinases (MMPs) are zinc-containing endopeptidases capable of destroying the components of the extracellular matrix, as well as activating many biologically active molecules (interleukins, growth factors, etc.) (23). Substrate specificity of various metalloproteinases is quite diverse, MMP-2 and MMP-9 are involved in the cleavage of denatured collagen (gelatin), elastin, and type IV collagen (24, 25). Insulin or insulin-like growth factor-1 signaling, through the PI3K/Akt cascade, regulate MMPs in different ways according to the target organ (23, 26).

Various studies reported varied results concerning MMPs expression profile, which can be related to the high heterogeneity of WAT (27, 28). But the majority of the results evidence that increased expression of MMPs leads to different complications in individuals with obesity with T2D. Induction of MMPs during adipocyte differentiation suggests that these enzymes may promote adipogenesis (29).

In our study, the MMPs expression was higher in T2D– patients with obesity, and such results are consistent with the study of Kosmala et al., who observed increased MMP-2 and MMP-9 activity in obese non-diabetic patients; herewith such processes were not observed in obese diabetic patients (28). We suppose that upregulated MMPs may be linked with the differentiation of adipose-derived stromal cells (ASCs) and the process of fibrosis.

SMAD4 and RUNX2, which are components of TGFβ signals, became the second group of genes that increased their expression in adipose tissue of T2D– patients with obesity. High level of the expression of these genes corresponded with decreased expression of mir-204-5p, mir-125b-5p, mir-125a-5p. The essence of activation of both TGFβ- and VMR-dependent signaling cascades is the activation of corresponding receptors with subsequent phosphorylation of SMAD proteins of different types (30). For TGFβ-signaling, it is predominantly SMAD2/3, and for BMP-signaling, it is SMAD 1/5/8. The activated SMADs bind to the heterodimeric complex with SMAD4, after which the nuclear translocation of the complex occurs, resulting in the stimulation of the expression and subsequent activation of the transcription factor RUNX2 and the expression of target genes (30).

However, according to Human Protein Atlas, neither SMAD4 nor RUNX2 is detected as protein products in adipose tissue (31). mRNA of these genes is detected in adipose tissue but at a very low level of RUNX2. All this allows us to assume that these genes are actually expressed in the composition of adipose tissue but in a certain minor cell population. Such a population may be a population of ASCs, and both SMAD4 and RUNX2 expression is shown in this population (32, 33). One of the essential properties of ASCs is their ability to differentiate in three main directions: adipogenic, osteogenic, and chondrogenic. In adipose tissue, ASCs are a population of progenitor cells that supports the process of natural adipocyte regeneration by means of adipogenic differentiation. In turn, RUNX2 is a transcription master regulator of osteogenic differentiation of mesenchymal precursors, and SMAD4 is a shuttle-protein for conducting SMADs in heterodimers to RUNX2 (34, 35). SMAD4-RUNX2 signal cascade is a critical participant not only in osteogenic differentiation but also in the regulation of chondrocyte hypertrophy (36). Reciprocal antagonistic relations between adipogenesis and osteogenesis are now well-described. It can be assumed that the activation of SMAD4-RUNX2 signal cascade leads to the commutation of progenitor cells of adipose tissue in the osteogenic direction (37).

The simultaneous increase of MMPs and SMAD4-RUNX2 genes in T2D– patients with obesity is consequent, as MMP9 presents a direct target of RUNX2. It has been shown that overexpression of RUNX2 significantly increases the endogenous levels of MMP9 by directly binding to a functional Runx2 regulatory element (38) MMPs genes and genes coding components of TGFβ signals refer to pro-fibrotic group regulating basement membrane remodeling. MMP-2 and MMP-9, along with MMP-14, modulate the tight pericellular collagens to allow preadipocytes to grow out of the stroma. Sun et al. showed on the mouse model that the absence of MMP-14 gene leads to the development of soft tissue fibrosis (39). Clinical studies also showed that the excess collagen limits healthy growth of adipose tissue and is linked with insulin resistance (40, 41). Thus, our transcriptional profiles confirm the severity and related metabolic complications of fibrosis in obese T2D+ individuals. But one of the limitations of our study is the absence of microscopic observation and immunohistochemistry of WAT fibrosis. These indicators were not taken into account in the design of the project. But based on the received data, we will include such criteria into the subsequent studies to achieve comprehensive results.

The second limitation lies in cell heterogeneity of WAT. It consists not only of adipocytes but also fibroblasts, endothelial cells, immune cells, and preadipocytes (5). Moreover, there is also heterogeneity within WAT adipocytes. Within subcutaneous depot, abdominal preadipocytes express higher pro-adipogenic marker PPARγ, which are more susceptible to apoptosis and are smaller in size due to increased lipolysis compared to gluteofemoral subcutaneous fat (42). Such heterogeneity, to a certain extent, reflects in transcription profiles leading to interpretation biases.

The present study is cross-sectional and do not give exact evidence of causality of obesity complications, however, it provides potentials target for drug development against T2D in individuals with obesity. Comprehension of the transcriptome difference between T2D+ and T2D– patients with obesity will allow to select the groups with high and low risk, and to determine the cohort of individuals, requiring the most intensive observation and treatment.

## Conclusion

The question of differences in pathological processes between individuals with obesity with the same BMI remains open. There is a growing body of evidence, that the key role in these cases belongs to miRs, as an important regulator of adipocyte metabolism. Based on the differential expression of specific miRs and mRNAs, we hypothesize that the main difference between T2D+ and T2D– patients with obesity consisted of adipogenesis regulation and prevailing of fibrosis processes in T2D+ patients with obesity. For further investigation of this problem, it is necessary to extend the patients cohorts and to engage cell and animal models.

## Data Availability Statement

The datasets generated for this study can be found in the Sequence Read Archive (SRA) PRJNA565427 (https://www.ncbi.nlm.nih.gov/sra/PRJNA565427).

## Ethics Statement

The studies involving human participants were reviewed and approved by the Endocrinology Research Center ethics committee (Protocol No. 9, 10.09.2017), Endocrinology Research Center, Moscow, Russia. The patients/participants provided their written informed consent to participate in this study.

## Author Contributions

AN, DK, ES, ISk, OB, AP, MM, AK, AY, VF, and YY: data collection, analysis, and interpretation. AN, ES, OB, ISt, and MS: study conception and design. AN and MS have interpreted the data and reviewing manuscript. ISk and OB drafting and critical revision of the manuscript.

### Conflict of Interest

The authors declare that the research was conducted in the absence of any commercial or financial relationships that could be construed as a potential conflict of interest.

## Supplementary Material

Figure S1 Heatmap diagram. Rows represent genes, and columns represent samples. Red, upregulated genes; blue, downregulated genes; white, unchanged gene expression. The diagram includes target genes for miRNAs.

Table S1 Prescribed medications for OBIR patients.

Table S2 Differential expression miRNAs (comparison MHO vs. MAO, adjusted *p*-value <0.05 and mean of normalized counts > 10).

Table S3 Differential expression mRNAs (comparison MHO vs. MAO, adjusted *p*-value <0.05) filtered by dysregulated miRNA from Table 2.

Table S4 Enrichment of important biological pathways among miRNAs differentially expressed between OBIS and OBIR patients by regulated genes.

## Abbreviations

ASCs: adipose-derived stromal cells
BAT: brown adipose tissue
BMI: body mass index
IGF-1R: insulin-like growth factor 1 receptor
IR: insulin resistance
miR: microRNA
MMPs: matrix metalloproteinases
RT-qPCR: reverse transcription-quantitative PCR
RUNX2: Runt-related transcription factor 2
SMADs: a family of structurally similar proteins that are the main signal transducers for receptors of the transforming growth factor beta
T2D: type 2 diabetes
TGFβ: Transforming growth factor beta
WAT: white adipose tissue.
