# Supplementary figures and images for: Role of MicroRNAs in the Regulation of Subcutaneous White Adipose Tissue in Individuals With Obesity and Without Type 2 Diabetes
Source: Front Endocrinol (Lausanne). 2019 Dec 5;10:840. doi: 10.3389/fendo.2019.00840 (PMC6906587; doi:10.3389/fendo.2019.00840)

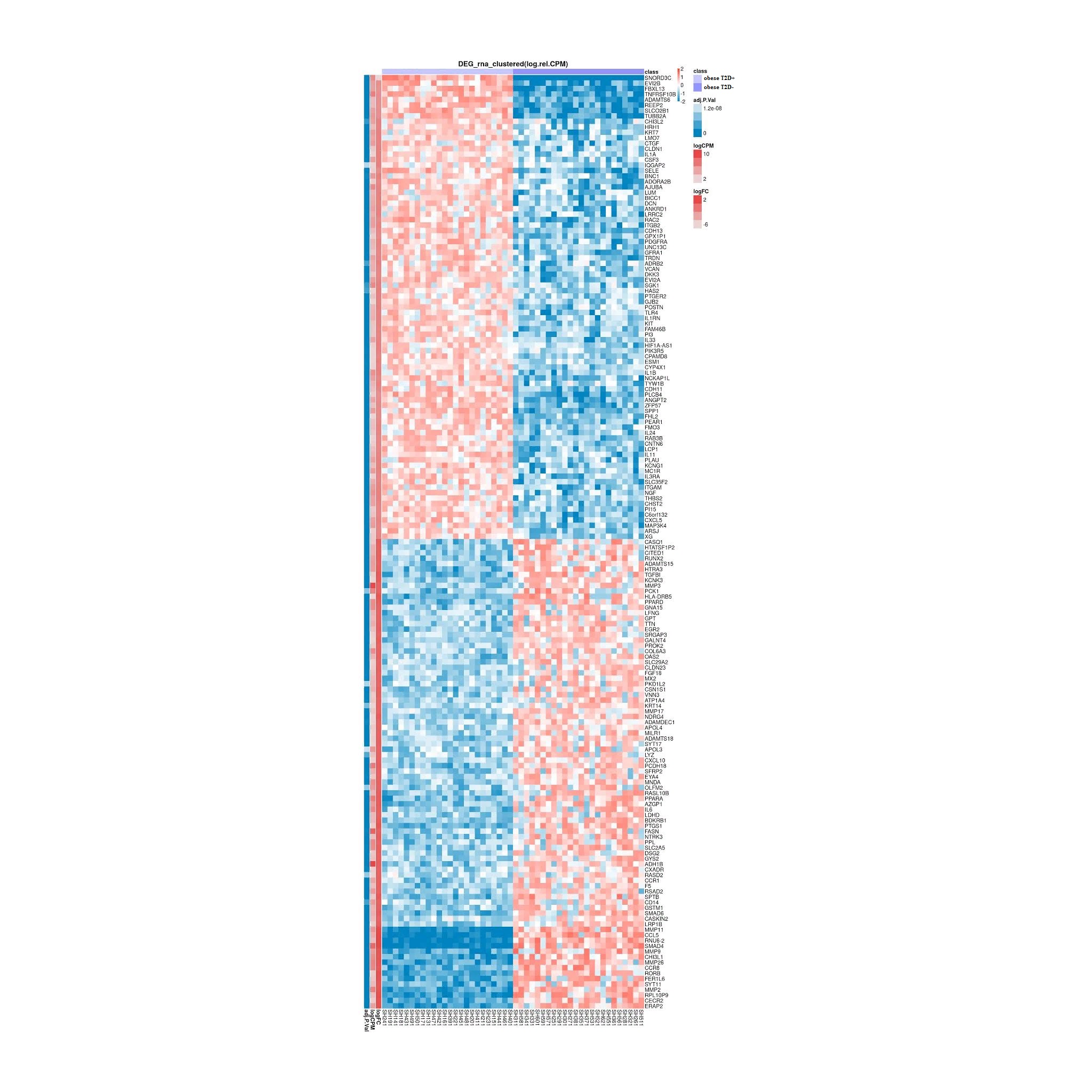

Supplement: Figure S1 — Heatmap diagram. Rows represent genes, and columns represent samples. Red, upregulated genes; blue, downregulated genes; white, unchanged gene expression. The diagram includes target genes for miRNAs. [file Image_1.JPEG]
