# Supplementary material for: Role of MicroRNAs in the Regulation of Subcutaneous White Adipose Tissue in Individuals With Obesity and Without Type 2 Diabetes
Source: Front Endocrinol (Lausanne). 2019 Dec 5;10:840. doi: 10.3389/fendo.2019.00840 (PMC6906587; doi:10.3389/fendo.2019.00840)
Supplement: Table S1 — Prescribed medications for OBIR patients. [file Table_1.DOCX]

Table S1. Prescribed medications for OBIR patients

| **Patient** | **Medication** | **Type of therapy** |
| --- | --- | --- |
| 1 | Metformin (1) | Two-component |
|  | Farxiga (2) |  |
| 2 | Metformin (1) | Two-component |
|  | Galvus (3) |  |
| 3 | Metformin (1) | Three-component |
|  | Galvus (3) |  |
|  | Diabeton (4) |  |
| 4 | Diabeton (4) | Monotherapy |
| 5 | Januvia (3) | Two-component |
|  | Farxiga (2) |  |
| 6 | Maninil (4) | Three-component |
|  | Metformin (1) |  |
|  | Jardiance (2) |  |
| 7 | Metformin (1) | Three-component |
|  | Galvus (3) |  |
|  | Lantus (5) |  |
| 8 | Metformin (1) | Monotherapy |
| 9 | Metformin (1) | Two-component |
|  | Diabeton (4) |  |
| 10 | Glickwidon (4) | Two-component |
|  | Metformin (1) |  |
| 11 | Metformin (1) | Three-component |
|  | Diabeton (4) |  |
|  | Farxiga (2) |  |
| 12 | Jardiance (2) | Monotherapy |
| 13 | Metformin (1) | Two-component |
|  | Januvia (3) |  |
| 14 | Metformin (1) | Three-component |
|  | Galvus (3) |  |
|  | Farxiga (2) |  |
| 15 | Maninil (4) | Three-component |
|  | Metformin (1) |  |
|  | Levemir (5) |  |
| 16 | Metformin (1) | Two-component |
|  | Onglyza (3) |  |
| 17 | Metformin (1) | Two-component |
|  | Galvus (3) |  |
| 18 | Metformin (1) | Monotherapy |
| 19 | Metformin (1) | Two-component |
|  | Galvus (3) |  |
| 20 | Metformin (1) | Two-component |
|  | Januvia (3) |  |
| 21 | Diabeton (4) | Monotherapy |
| 22 | Metformin (1) | Three-component |
|  | Farxiga (2) |  |
|  | Lantus (5) |  |
| 23 | Metformin (1) | Two-component |
|  | Lantus (5) |  |
| 24 | Metformin (1) | Three-component |
|  | Vipidia (3) |  |
|  | Jardiance (2) |  |
| 25 | Januvia (3) | Monotherapy |
| 26 | Metformin (1) | Three-component |
|  | Lantus (5) |  |
|  | Farxiga (2) |  |
| 27 | Metformin (1) | Monotherapy |
|  | Diabeton (4) |  |
| 28 | Diabeton (4) | Two-component |
|  | Farxiga (2) |  |
| 29 | Metformin (1) | Three-component |
|  | Galvus (3) |  |
|  | Farxiga (2) |  |
| 30 | Maninil (4) | Three-component |
|  | Metformin (1) |  |
|  | Lantus (5) |  |
| 31 | Metformin (1) | Two-component |
|  | Galvus (3) |  |
| 32 | Metformin (1) | Two-component |
|  | Jardiance (2) |  |
| 33 | Diabeton (4) | Monotherapy |
| 34 | Metformin (1) | Two-component |
|  | Glibenclamide (4) |  |
| 35 | Metformin (1) | Three-component |
|  | Januvia (3) |  |
|  | Maninil (4) |  |
| 36 | Metformin (1) | Monotherapy |

*****1 – Metformin; 2 – SGLT-2 inhibitors; 3 – DPP-4 inhibitors; 4 – sulfonylureas; 5 - insulin
