# Supplementary material for: Role of MicroRNAs in the Regulation of Subcutaneous White Adipose Tissue in Individuals With Obesity and Without Type 2 Diabetes
Source: Front Endocrinol (Lausanne). 2019 Dec 5;10:840. doi: 10.3389/fendo.2019.00840 (PMC6906587; doi:10.3389/fendo.2019.00840)
Supplement: Table S2 — Differential expression miRNAs (comparison MHO vs. MAO, adjusted p-value <0.05 and mean of normalized counts > 10). [file Table_2.docx]

Table S2. Differential expression miRNAs (comparison MHO vs MAO, adjusted p-value <0.05 and mean of normalized counts >10).

| **NGS** | | | | | **qPCR** | | |
| --- | --- | --- | --- | --- | --- | --- | --- |
| **MicroRNA (miR) symbol** | **Mean of normalized counts (rounded)** | **log_2_-FC** | **p-value** | **adjusted p-value** | **FC (95% confidence interval)** | **P** | **Q** |
| hsa-miR-1271-5p | 24 | -2,00 | 1,47E-10 | 1,46E-08 | Not measured | | |
| hsa-miR-501-3p | 102 | -1,48 | 3,32E-09 | 1,75E-07 | Not measured | | |
| hsa-miR-125a-5p | 27497 | -1,36 | 3,41E-08 | 1,33E-06 | **0.51 (0.38-0.75)** | **<0.001** | **0.001** |
| hsa-miR-145-3p | 26 | -1,29 | 6,89E-08 | 2,20E-06 | Not measured | | |
| hsa-miR-6803-3p | 35 | -1,18 | 1,51E-07 | 4,45E-06 | Not measured | | |
| hsa-miR-140-3p | 741 | -1,37 | 5,08E-07 | 1,17E-05 | 0.67 (0.40-1.02) | 0.075 | 0.990 |
| hsa-let-7e-3p | 39 | -1,17 | 6,71E-07 | 1,46E-05 | 1.22 (0.81–1.74) | 0.090 | 0.963 |
| hsa-miR-193b-3p | 1242 | -1,48 | 8,44E-07 | 1,80E-05 | 0.74 (0.42-1.06) | 0.090 | 0.960 |
| hsa-miR-3656 | 84 | -1,19 | 2,11E-06 | 3,84E-05 | Not measured | | |
| hsa-miR-20a-5p | 26 | 1,26 | 2,74E-06 | 4,62E-05 | Not measured | | |
| hsa-miR-320d | 22 | -1,15 | 5,72E-06 | 8,99E-05 | Not measured | | |
| hsa-miR-197-3p | 7053 | 1,01 | 9,13E-06 | 0,00012774 | **1.47 (0.92–1.98)** | **<0.001** | **0.02** |
| hsa-miR-485-3p | 24 | 1,40 | 9,38E-06 | 0,00012917 | 1.01 (0.76–1.39) | 0.491 | 1 |
| hsa-miR-27a-3p | 74 | -1,40 | 1,06E-05 | 0,00014388 | Not measured | | |
| hsa-miR-23b-3p | 429 | 1,31 | 1,24E-05 | 0,00016305 | **2.75 (2.01–3.56)** | **<0.001** | **0.012** |
| hsa-miR-365b-5p | 95 | -1,50 | 1,29E-05 | 0,00016711 | Not measured | | |
| hsa-miR-1233-3p | 12 | -1,03 | 1,33E-05 | 0,00016985 | Not measured | | |
| hsa-miR-654-3p | 47 | 1,22 | 1,73E-05 | 0,00020659 | Not measured | | |
| hsa-miR-204-5p | 1107 | -1,67 | 1,93E-05 | 0,00021278 | **0.18 (0.08-0.28)** | **<0.001** | **<0.001** |
| hsa-miR-320a | 4127 | -1,15 | 1,95E-05 | 0,00021278 | **0.50 (0.34–0.77)** | **<0.001** | **0.015** |
| hsa-miR-511-5p | 15 | -1,26 | 1,97E-05 | 0,00021284 | Not measured | | |
| hsa-miR-107 | 291 | -1,20 | 2,20E-05 | 0,00023176 | 0.96 (0.71-1.23) | 0.266 | 1 |
| hsa-miR-483-5p | 92 | -1,05 | 2,37E-05 | 0,0002414 | Not measured | | |
| hsa-miR-98-5p | 1541 | -1,13 | 5,24E-05 | 0,00045288 | 0.95 (0.69–1.27) | 0.801 | 1 |
| hsa-miR-502-3p | 29 | -1,09 | 5,68E-05 | 0,00047987 | Not measured | | |
| hsa-miR-1910-5p | 10 | -1,26 | 5,68E-05 | 0,00047987 | Not measured | | |
| hsa-miR-192-5p | 46 | -1,02 | 7,10E-05 | 0,00056735 | Not measured | | |
| hsa-miR-1275 | 16 | -1,13 | 7,93E-05 | 0,00062242 | Not measured | | |
| hsa-miR-375 | 17 | -1,30 | 8,64E-05 | 0,00067257 | Not measured | | |
| hsa-miR-432-5p | 47 | -1,12 | 9,26E-05 | 0,00071335 | Not measured | | |
| hsa-miR-1247-5p | 275 | -1,07 | 9,83E-05 | 0,00073939 | 0.94 (0.70-1.21) | 0.422 | 1 |
| hsa-miR-296-5p | 15 | -1,11 | 0,00014229 | 0,00098721 | Not measured | | |
| hsa-miR-99b-5p | 10114 | -1,01 | 0,0002011 | 0,0013042 | **0.42 (0.29-0.64)** | **<0.001** | **0.01** |
| hsa-miR-125b-5p | 4621 | -1,02 | 0,00021621 | 0,0013922 | **0.46 (0.31-0.65)** | **<0.001** | **0.01** |
| hsa-miR-122-5p | 99 | -1,71 | 0,002591 | 0,010171 | Not measured | | |
| hsa-miR-196a-5p | 11 | -1,01 | 0,0034576 | 0,012631 | Not measured | | |
