# Supplementary material for: Role of MicroRNAs in the Regulation of Subcutaneous White Adipose Tissue in Individuals With Obesity and Without Type 2 Diabetes
Source: Front Endocrinol (Lausanne). 2019 Dec 5;10:840. doi: 10.3389/fendo.2019.00840 (PMC6906587; doi:10.3389/fendo.2019.00840)
Supplement: Table S3 — Differential expression mRNAs (comparison MHO vs. MAO, adjusted p-value <0.05) filtered by dysregulated miRNA from Table 2. [file Table_3.docx]

Table S3. Differential expression mRNAs (comparison MHO vs MAO, adjusted p-value <0.05) filtered by dysregulated miRNA from table 2.

| **NGS** | | | | | **qPCR** | | |
| --- | --- | --- | --- | --- | --- | --- | --- |
| **Gene symbol** | **log_2_-FC** | **log_2_-CPM** | **p-value** | **adjusted p-value** | **FC (95% confidence interval)** | **P** | **Q** |
| MMP3 | 1.0949 | 11.04 | 2.645e-15 | 2.802e-13 | 2.01 (1.27–2.77) | 0.01 | 0.2 |
| MAP3K4 | -1.3832 | 5.0522 | 3.3406e-22 | 6.7082e-20 | 0.97 (0.71–1.22) | 0.851 | 1 |
| **MMP9** | 1.9675 | 5.0787 | 1.0279e-30 | 4.6098e-28 | **1.92 (1.51–2.31)** | **< 0.001** | **0.004** |
| LCP1 | -1.4863 | 2.5819 | 1.9456e-24 | 5.0339e-22 | 0.92 (0.72–1.11) | 0.305 | 1 |
| SLCO2B1 | -2.2529 | 4.6522 | 2.6046e-47 | 2.503e-44 | 1.05 (0.80–1.31) | 0.535 | 1 |
| CCR8 | 1.8177 | 6.5563 | 2.8466e-34 | 1.6651e-31 | 1.29 (0.97–1.67) | 0.002 | 0.051 |
| TNFAIP8 | -3.1689 | -0.9217 | 1.0596e-08 | 3.108e-07 | 0.79 (0.52–1.06) | 0.119 | 1 |
| IL6 | 1.3176 | 6.4039 | 7.7424e-16 | 8.7535e-14 | 5.02 (1.79–8.55) | 0.019 | 0.272 |
| **MMP2** | 2.0971 | 6.9353 | 1.6109e-49 | 1.8509e-46 | **1.29 (1.11–1.46)** | **0.002** | **0.042** |
| **MMP26** | 2.0712 | 6.8292 | 8.4676e-45 | 7.5949e-42 | **1.29 (1.11–1.46)** | **0.002** | **0.042** |
| **MMP11** | 2.85 | 5.4909 | 2.7054e-75 | 7.2798e-72 | **1.44 (1.22–1.67)** | **< 0.001** | **0.007** |
| PPARD | 1.4987 | 6.2344 | 1.0399e-23 | 2.5439e-21 | 1.07 (0.85–1.30) | 0.424 | 1 |
| TLR4 | -1.1747 | 3.3433 | 1.7196e-14 | 1.6765e-12 | 0.77 (0.47–1.09) | 0.129 | 1 |
| PPARA | 1.521 | 7.3269 | 2.1423e-23 | 5.0565e-21 | 1.57 (0.40–2.77) | 0.305 | 1 |
| SPP1 | -1.8063 | 4.8456 | 4.8303e-40 | 3.2493e-37 | 0.61 (0.31–0.95) | 0.02 | 0.264 |
| **SMAD4** | 3.0695 | 8.8106 | 6.8842e-90 | 2.3155e-86 | **3.24 (1.98–4.50)** | **0.002** | **0.040** |
| TNFRSF10B | -2.1865 | 7.1604 | 1.2252e-48 | 1.268e-45 | 0.91 (0.57–1.29) | 0.621 | 1 |
| MNDA | 1.1954 | 5.9417 | 4.2764e-20 | 7.2829e-18 | 1.61 (1.20–2.09) | 0.005 | 0.109 |
| SMAD6 | 1.7322 | 3.5973 | 1.1503e-29 | 4.8363e-27 | 1.74 (1.35–2.17) | 0.017 | 0.225 |
| NCKAP1L | -1.5855 | 4.8048 | 6.1667e-28 | 2.2423e-25 | 0.91 (0.55–1.29) | 0.621 | 1 |
| GNA15 | 1.0761 | 6.1117 | 3.4673e-13 | 2.7121e-11 | 1.49 (0.82–2.09) | 0.129 | 1 |
| RAC2 | -1.6411 | 4.7248 | 1.8157e-24 | 4.7899e-22 | 0.91 (0.71–1.16) | 0.495 | 1 |
| ITGAM | -1.1835 | 4.9845 | 7.0612e-14 | 6.051e-12 | 0.89 (0.69–1.07) | 0.19 | 1 |
| EVI2A | -1.3384 | 4.9029 | 1.7313e-24 | 4.6587e-22 | 0.74 (0.52–1.01) | 0.039 | 1 |
| EVI2B | -2.0751 | 4.7494 | 5.2879e-37 | 3.2338e-34 | 0.82 (0.55–1.17) | 0.324 | 1 |
| TUBB2A | -2.1838 | 5.2821 | 1.1921e-44 | 1.0024e-41 | 0.91 (0.64–1.27) | 0.646 | 1 |
| MC1R | -1.0712 | 4.9648 | 1.341e-14 | 1.3169e-12 | 0.79 (0.52–1.24) | 0.524 | 1 |
| KIT | -1.1804 | 2.064 | 4.7361e-23 | 1.08e-20 | 0.71 (0.49–1.09) | 0.129 | 1 |
| **RUNX2** | 1.297 | 4.6497 | 2.011e-17 | 2.7329e-15 | **1.5 (1.29–1.70)** | **< 0.001** | **0.001** |
| FBXL13 | -2.4842 | 4.6851 | 1.2466e-51 | 2.0965e-48 | 0.95 (0.70–1.22) | 0.596 | 1 |
| ITGB2 | -1.2392 | 4.8629 | 5.3749e-16 | 6.2882e-14 | 0.91 (0.72–1.19) | 0.212 | 1 |
