# Supplementary material for: Role of MicroRNAs in the Regulation of Subcutaneous White Adipose Tissue in Individuals With Obesity and Without Type 2 Diabetes
Source: Front Endocrinol (Lausanne). 2019 Dec 5;10:840. doi: 10.3389/fendo.2019.00840 (PMC6906587; doi:10.3389/fendo.2019.00840)
Supplement: Table S4 — Enrichment of important biological pathways among miRNAs differentially expressed between OBIS and OBIR patients by regulated genes. [file Table_4.DOCX]

Table S4. Enrichment of important biological pathways among miRNAs differentially expressed between OBIS and OBIR patients by regulated genes.

| **Reactome annotation** | **p-Value** | **miRNAs** |
| --- | --- | --- |
| Gene Expression | 7.55e-16 | \| hsa-miR-125a-5p \| ↓ \| \| --- \| --- \| \| hsa-miR-197-3p \| ↑ \| \| hsa-miR-23b-3p \| ↑ \| \| hsa-miR-204-5p \| ↓ \| \| hsa-miR-320a \| ↓ \| \| hsa-miR-99b-5p \| ↓ \| \| hsa-miR-125b-5p \| ↓ \| |
| Translation | 3.09e-7 |  |
| Generic Transcription Pathway | 4.61e-7 |  |
| Eukaryotic Translation Initiation | 4.61e-7 |  |
| Cap-dependent Translation Initiation | 4.61e-7 |  |
| Cellular responses to stress | 5.36e-7 |  |
| 3' -UTR-mediated translational regulation | 0.00000656 |  |
| Eukaryotic Translation Elongation | 0.0000289 |  |
| Eukaryotic Translation Termination | 0.0000395 |  |
| Activation of Matrix Metalloproteinases | 0.00252 |  |
